# A case of maternal varicella: Expected role of primary care physicians in confirming immune status for varicella in women at childbearing age

**DOI:** 10.1002/jgf2.291

**Published:** 2019-12-13

**Authors:** Yuji Nishihara, Ryota Hase

**Affiliations:** ^1^ Department of Infectious Diseases Japanese Red Cross Narita Hospital Narita Chiba Japan; ^2^ Department of Infectious Diseases Kameda Medical Center Kamogawa Chiba Japan

**Keywords:** maternal varicella, preconception counseling, varicella‐zoster virus

## Abstract

At 19‐week gestation, a 32‐year‐old Japanese woman presented with a 3‐day history of fever and vesicular rashes on the skin and was diagnosed with varicella, which resolved after antiviral therapy. In the primary clinic, her immunity to rubella and measles, but not to varicella, was confirmed at preconception counseling. Maternal varicella infection can cause congenital varicella syndrome characterized by congenital malformations and neurological deficits. This case recommends that all women of childbearing age should be assessed for immunity to varicella before pregnancy and that primary care physicians should take initiatives for preventing maternal varicella.

## Graphical abstract

Maternal varicella infection can cause congenital varicella syndrome. All women of childbearing age should be assessed for immunity to varicella before pregnancy.

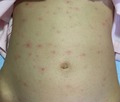

## 1 INTRODUCTION

Varicella is one of the TORCH infections that can result in stillbirth, perinatal morbidity, and severe sequelae by transmission from mothers to their children.

Although primary care physicians are aware of the risk of rubella during pregnancy, the risk of varicella‐zoster virus (VZV) is underestimated. The antibody titer of rubella is typically measured at the preconception care visit; however, immunity to varicella is not typically checked. Here, we describe the case of a 32‐year‐old Japanese woman at 19‐week gestation who was diagnosed with varicella. Her immunity to rubella and measles, but not to varicella, was confirmed at preconception counseling.

## 2 CASE REPORT

The patient was 32‐year‐old Japanese woman at 19‐week gestation who reported no history of relevant illness. Before trying to conceive, she had preconception counseling with her primary care physician, where her rubella and measles antibodies were confirmed to be positive. Although she had no previous history of varicella or vaccination, neither were her antibodies to VZV checked nor was a vaccination provided at preconception counseling.

In 3 days before admission, she had general malaise, headache, and a vesicular rash on her chest. On the day of admission, she visited a nearby hospital because the rash was spreading all over her body. She was suspected of varicella and referred to the Japanese Red Cross Narita Hospital.

**Figure 1:**
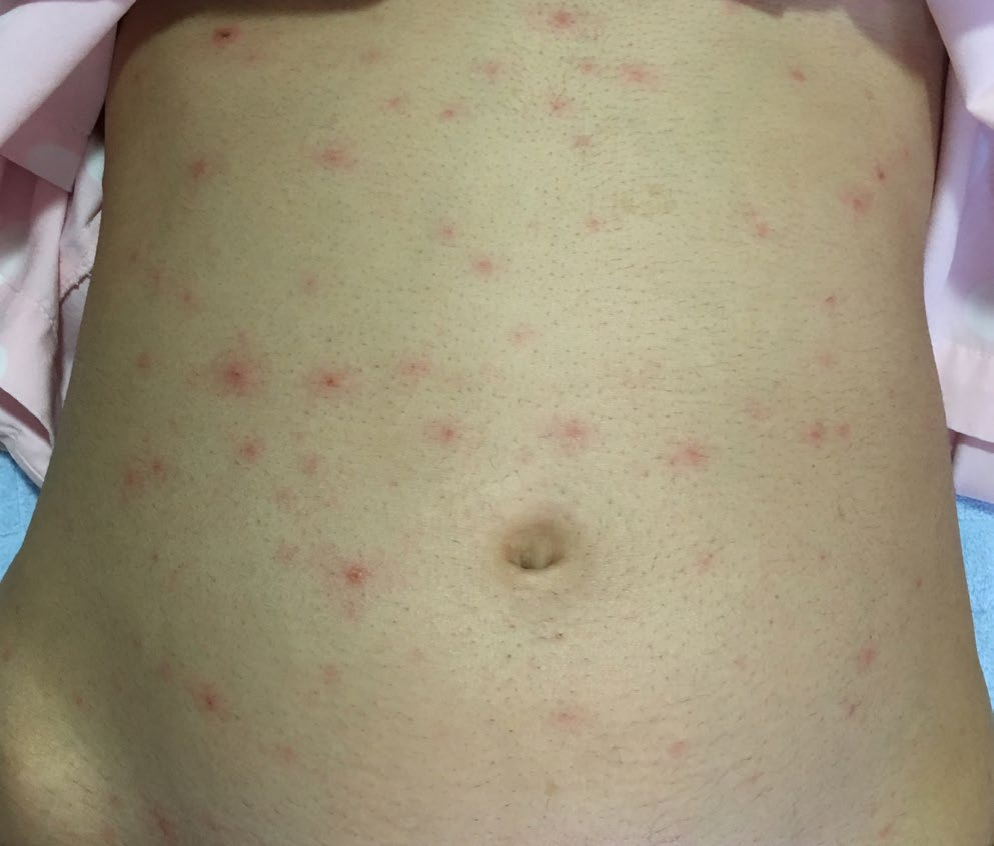
Picture of the patient's skin upon admission. The rash includes macular, papular, vesicular, and crusted lesions in different stages

On admission, her consciousness was clear and vital signs were as follows: blood pressure, 120/74 mm Hg; heart rate, 88 beats/min; body temperature, 37.4°C; respiratory rate, 15 breaths/min; and oxygen saturation on room air, 97%. Physical examination was notable for vesicular rashes and macules and papules on the face, trunk, and extremities (Figure 1). No other signs of complications, including pneumonia, were noted.

Laboratory studies revealed a white blood cell count of 60 × 10^2^/µL, hemoglobin level of 12.8 g/dL, platelet count of 120 000 cells/L, serum creatinine level of 0.44 mg/dL, AST level of 88 IU/L, ALT level of 80 IU/L, and C‐reactive protein level of 1.83 mg/dL.

The patient was clinically diagnosed with varicella based on the characteristic of generalized vesicular rashes. She was admitted to the hospital due to poor oral intake and started on intravenous acyclovir at 10 mg/kg per dose three times a day. To confirm the diagnosis of varicella, the VZV antibody was measured and polymerase chain reaction (PCR) of VZV for whole blood and vesicular exudate was performed. On hospital day 5, her medication was switched to oral acyclovir 800 mg five times a day. She completely recovered and was discharged after 7 days of acyclovir treatment. The final diagnosis was confirmed both by positive PCR of whole blood and vesicular exudate and paired serology (IgM 1.03, IgG < 2.0 measured upon admission, IgM 5.53, IgG 37.2 after 3 weeks). After discharge, she delivered a healthy infant at 37 weeks of gestation. No congenital deficit was observed at birth by the pediatrician.

## 3 DISCUSSION

The present case reports the importance of preconception immunization against not only rubella but also varicella and highlights the important role of primary care physicians in preventing maternal varicella in mothers of childbearing age.

Varicella during pregnancy is characterized by increased maternal mortality and severe complications. The mortality rate of varicella in pregnancy is five times higher than that in nonpregnant adults.^1^ Varicella pneumonia is one of the most common complications in pregnant women. A study on 43 pregnancies with varicella revealed that 9% of cases developed varicella pneumonia.^2^

In addition to serious impacts on the mother, varicella infection also has teratogenic effects to the fetus. Varicella infection in the first two trimesters carries a risk of congenital varicella syndrome (CVS) with a frequency of 0.4%–2% (first trimester: 0.4% and second trimester: 2%). The major characteristics of CVS include skin scarring, limb hypoplasia, chorioretinitis, and microcephaly.^3^

The risk of congenital varicella tends to be underestimated more than that of congenital rubella. Accordingly, immunity to VZV is not commonly checked at preconception care. Although not specifically referring to varicella, the Japan Society of Obstetrics and Gynecology recommends confirming immunity to rubella before infertility treatment.^4^ Rubella is well known for causing fetal abnormalities in women who are infected during pregnancy. The virus passes from the mother to the fetus with a frequency of 90% in the first trimester, and majority of infected infants may develop congenital rubella syndrome (CRS) marked by congenital heart defects, cataracts, and sensorineural defects.^5^ Although varicella carries a lower risk of fetal infection compared with rubella, infant mortality does not differ between CRS and CVS. A study found that the mortality rate in symptomatic infants with CRS was approximately 20%,^6^ whereas 30% of infants with CVS died during the first few months of life.^7^ In the present case, immunity to rubella and measles, but not to varicella, was checked by the primary care physician at preconception counseling. The primary care physician should have evaluated for immunity to varicella and vaccinated the patient. Fortunately, the infant in the present case did not develop CVS.

Because the varicella vaccine is a live vaccine and cannot be given to pregnant women, appropriate assessment for immunity to varicella should be performed in all women of childbearing age before pregnancy. Evidence of immunity to varicella can be assessed using vaccination records, verification by a healthcare provider, or a positive antibody. A two‐dose VZV vaccination has 98% efficacy against varicella infection,^8^ and women without proven immunity to varicella should receive these vaccines.

This strategy would be particularly important in the next a few decades in Japan in the context of paradoxical effect. Varicella vaccination was introduced into Japan's routine vaccination program in October 2014, resulting in the drastic decrease in varicella patients.^9^ In addition, surveillance data for seroprevalence of antibodies to varicella in Japanese population between 2014 and 2017 have revealed that the seropositivity rate for varicella is approximately ≥90% at childbearing age.^10^ However, the suppression of natural infection leads to a low opportunity of acquiring natural immunity. As a result, women born before the vaccination program started have less chances of acquiring immunity to varicella, resulting in an increase in their susceptibility to residual exposures during later pregnancy stages, called “paradoxical effect.^11^” Therefore, appropriate catch‐up vaccination policy should be considered for this generation to minimize the risk of this paradoxical effect.

Primary care physicians play an important role in preconception care and have a responsibility to confirm the immune status of vaccine‐preventable diseases and appropriately vaccinate. Although most women have their first prenatal visit at ≥8 weeks of pregnancy,^12^ the timing is too late to provide live vaccinations such as rubella, measles, or varicella. Additionally, because some pregnancies are unintended, primary care physicians should consider every woman of childbearing age as a candidate for preconception care. The American Academy of Family Physicians recommends preconception counseling and care including the administration of vaccines for measles, mumps, rubella, and varicella, to all nonpregnant, nonimmune women.^13^

Obstetricians and gynecologists can also assess immunity against varicella for pregnant women when the evaluation has not been performed before pregnancy. They should be responsible for providing varicella vaccination to pregnant women having insufficient immunity after delivery. Although the guideline in UK does not recommend universal serological antenatal testing,^14^ we believe that assessing the evidence of immunity to varicella for pregnant women would be valuable in the next few decades in Japan in the context of paradoxical effect.

In conclusion, we reported the case of a pregnant patient with varicella whose immune status was not evaluated for VZV at preconception care. All women of childbearing age should be evaluated for immunity not only to rubella but also to VZV before pregnancy. Primary care physicians should confirm immune status for varicella in women of childbearing age in their daily practices.

## CONFLICT OF INTEREST

The authors have stated explicitly that there are no conflicts of interest in connection with this article.

## INFORMED CONSENT

We have obtained written informed consent from the patient for publication of this case report.
